# Lived Experiences of Male Caregivers Supporting Individuals with Parkinson’s Disease

**DOI:** 10.3390/healthcare14121652

**Published:** 2026-06-11

**Authors:** Kelsey Lee, Julia Yates, Andrew M. Johnson, Liliana Alvarez, Jeffrey D. Holmes

**Affiliations:** 1 School of Occupational Therapy, Western University, London, ON N6A 3K7, Canada; klee729@uwo.ca (K.L.); lalvare2@uwo.ca (L.A.); 2 Health and Rehabilitation Sciences, Western University, London, ON N6A 3K7, Canada; jyates23@uwo.ca; 3 School of Health Studies, Western University, London, ON N6A 3K7, Canada; ajohnson@uwo.ca

**Keywords:** Parkinson’s disease, male caregivers, gender, caregiving, caregiver burden

## Abstract

**Background/Objectives:** Individuals living with Parkinson’s disease often rely on informal caregivers for daily support. Existing research on caregiving has largely been framed from a predominantly female perspective. However, caregiving roles are evolving, with an increasing number of men assuming these responsibilities. Despite this shift, the experiences of male caregivers remain underexplored. A deeper understanding of their perspectives is essential to inform more inclusive and effective caregiver support in Parkinson’s disease care. This study aimed to explore the lived experience of male caregivers supporting individuals with Parkinson’s disease. **Methods:** A secondary qualitative analysis was conducted using interviews with seven male caregivers supporting individuals with Parkinson’s disease. Data were analyzed using thematic analysis within a descriptive phenomenological framework. **Results:** Three overarching themes emerged. (1) Economic and employment challenges, including difficulties balancing work and caregiving, structural barriers to accessing services during working hours, and the financial burden of Parkinsons-related expenses, with financial stability acting as a buffer against stress; (2) Psychological and emotional dynamics, including self-doubt linked to gender norms, the influence of personal and partner attitudes on coping, and ongoing experiences of loss; and (3) Adaptation and coping, involving renegotiation of roles, maintenance of routines, and information seeking that was experienced as both empowering and anxiety-provoking. **Conclusions:** Male caregiving experiences were shaped by financial stability, personal beliefs and perspectives, and the ability to adapt to changing roles and responsibilities. These findings highlight the importance of recognizing gender-specific caregiving needs and incorporating targeted support for male caregivers into Parkinson’s disease care.

## 1. Introduction

Parkinson’s disease (PD) is one of the fastest-growing neurological conditions worldwide, affecting more than 8.5 million people and projected to exceed 25 million cases by 2050 [1,2,3]. As the number of individuals with PD (IwPD) increases, so too does the reliance on informal caregivers for daily support. Many IwPD depend on family members or friends for assistance with everyday activities, and caregiving demands can be substantial, with some caregivers spending up to 16 h per day fulfilling these responsibilities [4]. Although caregiving can foster closer relationships and provide a sense of fulfillment, personal growth, and emotional reward [5,6,7], it is also associated with significant burden [8,9,10].

Caregiver burden encompasses physical, emotional, social, financial, and spiritual strain [5,6,9,11]. This burden is often exacerbated by the severity of PD symptoms, particularly non-motor symptoms such as anxiety and cognitive impairment [6,12,13,14,15]. Individual caregiver characteristics, including coping style, mental health, and access to social support, further influence the caregiving experience [5,9,13]. Higher levels of caregiver anxiety and depression, as well as limited social support, are associated with greater burden, whereas strong support networks may mitigate its effects [5,9,13]. Consequences of caregiver burden include persistent worry, guilt, reduced social engagement, restrictions in daily activities, and decreased life satisfaction [5,6,9,11].

PD is more prevalent among men, with the prevalence increasing from 1.56 to 6.44 million since 1990 [16], and women are more frequently identified as primary caregivers [9,17,18]. However, caregiving patterns are evolving across diverse family structures, with more men assuming caregiving responsibilities for their partners and the number of openly same-sex partnerships in Canada continuing to grow [19,20]. In these contexts, men may encounter unique challenges when adopting roles traditionally associated with women [18].

Despite these demographic and societal shifts, the PD caregiving literature remains heavily centered on female caregivers, leaving male caregivers comparatively underrepresented and poorly understood. In a recent scoping review by Longacre and colleagues [17], 31 studies were examined, although only 24 reported participant gender. Among those studies, women constituted the majority of participants in nearly all cases, with an average of 76.1% female representation. Only one study reported an equal number of male and female participants (50% each), while another identified a slight male majority of 51.43%. Existing studies examining gender differences in caregiving for IwPD are limited and inconclusive. Some report lower quality of life among female caregivers [21], while findings related to caregiver burden remain mixed and are often constrained by unequal gender representation and insufficient attention to men’s experiences [17,22]. Broader caregiving literature suggests that women tend to experience greater emotional and physical strain, perform more personal and household caregiving tasks, and report higher stress levels than men [9,23,24,25,26]. However, these findings are largely derived from heterogeneous caregiving populations and may not adequately reflect the specific realities of caregiving in PD or the experiences of male caregivers within this context.

Importantly, little is known about how male caregivers of IwPD experience and interpret caregiving roles, adapt to caregiving responsibilities, or navigate the emotional, relational, and social impacts of PD caregiving [17]. The limited representation of men in PD caregiving research has resulted in a significant gap in understanding the unique needs, challenges, and coping strategies of this population. Addressing this gap is essential to developing a more inclusive and gender sensitive understanding of caregiving in PD. Therefore, this study explores the lived experiences of male informal caregivers supporting IwPD, with the aim of informing more equitable caregiver research, support, and interventions.

## 2. Materials and Methods

### 2.1. Study Design

This qualitative study employed a cross-sectional design combining secondary and primary analyses to explore the distinctive experiences of male informal caregivers supporting IwPD. Initially, three male caregivers were interviewed as part of a broader study without specific questions about male caregiving experiences. During preliminary analysis, unique themes related to male caregiving emerged, prompting further investigation. Subsequently, four additional male caregivers were recruited and interviewed using an adapted guide that included questions specifically addressing their experiences as male caregivers. The study was conducted in accordance with the Declaration of Helsinki and approved by the Institutional Review Board of Western University (protocol #112490; 19 March 2019).

### 2.2. Recruitment and Eligibility

Participants were recruited from a database of individuals affiliated with a local chapter of the Parkinson Society. Written informed consent was obtained from all subjects involved in the study. To be eligible, participants: (a) self-identified as male; (b) were fluent in English; (c) were currently providing unpaid care to an IwPD or had been doing so within the previous 18 months; and (d) were able to participate in a semi-structured interview.

### 2.3. Data Collection

Seven male caregivers participated in semi-structured interviews across two phases of data collection. Three participants were interviewed as part of a broader study conducted in 2019, during which insights related to male caregiving were identified. To further explore the gender-specific experiences, four additional males were recruited and interviewed in 2024.

For both phases, initial interviews lasted approximately 60–90 min, and follow-up interviews lasted 30–45 min. Each initial interview began with participants sharing their personal journey as a caregiver to an IwPD, providing contextual grounding for the discussion. Interviews then followed a semi-structured format, focusing broadly on participants’ experiences of caregiving, including perceived burden and access to resources and supports. To elicit comprehensive information, participants were asked a series of semi-structured questions addressing: (1) how life had changed since assuming the caregiving role; (2) resources or supports accessed to assist in caring for the IwPD; (3) resources or supports accessed to support their own health and well-being; (4) supports specifically related to managing caregiver burden; (5) desired but inaccessible supports; and (6) perceived barriers to accessing such resources.

In the second phase of data collection, participants were provided with an additional prompt at the outset of the interview, inviting them to reflect on how being male may have influenced their caregiving experiences. This prompt was used to sensitize participants to potential gender-related dimensions of caregiving and to support more explicit discussion of these factors throughout the interview.

Follow-up interviews supported collaborative reflection to enhance the accuracy, resonance, credibility, and trustworthiness of the findings [27]. During these interviews, participants were presented with preliminary themes identified across the dataset and invited to offer feedback and additional insight. These interviews also provided an opportunity for participants to clarify and elaborate on interpretations arising from their initial interviews.

### 2.4. Data Analysis

**Figure 1:**
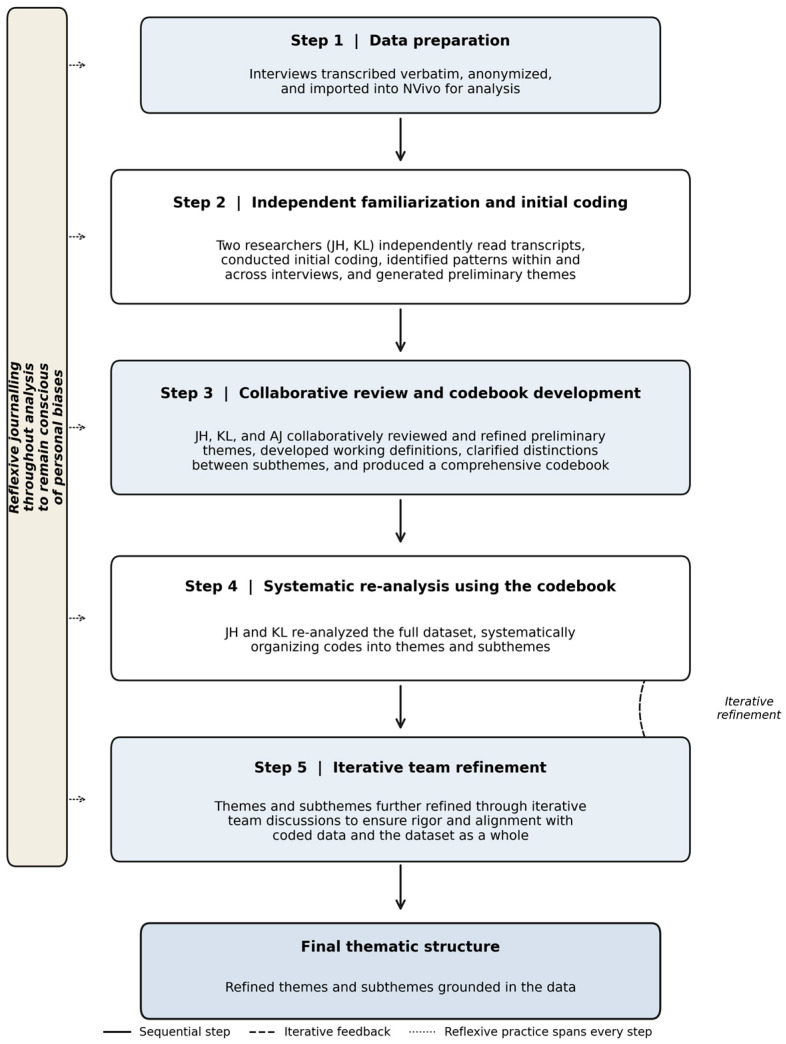
Data analysis process.

Interviews were transcribed verbatim, anonymized, and analyzed using inductive thematic analysis [28,29] in NVivo Version 14. Two researchers (JH, KL) independently familiarized themselves with the data by reading transcripts, conducting initial coding, and identifying initial patterns within and across interviews to generate preliminary themes. JH and KL then met with a third researcher (AJ) to collaboratively review and refine these preliminary themes. During this process, the team developed working definitions, clarified distinctions between subthemes, and produced a comprehensive codebook to guide subsequent analysis. Using this codebook, JH and KL re-analyzed the full dataset, systematically organizing codes into themes and subthemes [28,29]. These were further refined through iterative team discussions to ensure rigor and alignment with both the coded data and the dataset as a whole. Reflexive journalling was employed throughout to remain conscious of any personal biases (see Figure 1 for a schematic representation of the data analysis process).

To ensure study rigor, strategies were employed to support credibility, confirmability, dependability, and potential transferability [30]. Credibility was enhanced through iterative data collection and analysis, including the use of follow-up interviews in which participants reviewed and provided feedback on preliminary themes (i.e., member checking). These follow-up interviews also allowed for clarification and deeper exploration of participants’ experiences. Confirmability was supported through reflexive analytic practices, including ongoing team discussions and documentation of analytic decisions to ensure that interpretations remained grounded in participants’ accounts. Dependability was addressed by maintaining a transparent and systematic approach to data collection and analysis across both phases of the study, including the use of a consistent semi-structured interview guide and documentation of any adaptations made during the second phase. Transferability was supported through the inclusion of rich, verbatim participant quotations and detailed descriptions of caregiving experiences, enabling readers to assess the relevance of the findings to other contexts.

The qualitative data generated and analyzed during the study are not publicly available because they contain potentially identifiable participant information and participants did not consent to public data sharing. Additional information may be available from the corresponding author upon reasonable request and subject to institutional ethics approval. Sections of this manuscript were edited for language clarity using ChatGPT-5.3. No substantive changes were made to the content. The authors take full responsibility for the accuracy and integrity of the work.

## 3. Results

Participant demographic characteristics are presented in Table 1. Thematic analysis revealed three overarching themes and seven subthemes, outlined below.

### 3.1. Theme 1: Economic and Employment Challenges in Caregiving

This theme captures the economic dimensions of caregiving, including both financial pressures and the challenges of balancing employment with caregiving responsibilities. Experiences varied across participants. While some caregivers described significant strain related to combining paid work with caregiving, others reported fewer employment-related challenges, particularly when retired or financially stable. Across accounts, financial resources played an important role in shaping access to supports, with economic stability acting as a buffer against stress and financial constraints intensifying the burden of care. Two subthemes emerged within this broader theme.

#### 3.1.1. Balancing Act: The Stress of Juggling Employment and Caregiving Responsibilities

Some participants described challenges managing employment alongside caregiving responsibilities, particularly those working full-time. For these caregivers, the demands of paid work combined with caregiving responsibilities created strain on time, energy, and overall well-being. One participant conveyed the cumulative exhaustion associated with managing both roles, stating, “*I just feel tired all the time, I feel mentally swamped and mentally drained all the time. I work. I don’t have the patience for any of it because I feel like I’m never getting a break, because I’m never on my own just to relax, you know?*” (CG1). Similarly, another caregiver emphasized the complexity of navigating multiple social roles simultaneously, explaining, “*At the time, I was still working at my job, trying to juggle both the requirements of being a wife, a caregiver, my job and the back and forth… So that was part of the considerations and factors that went into deciding: When do I retire? How do you balance it all?*” (CG6). However, employment-related burden was not universal. Several participants were retired or had more flexible work arrangements, and these caregivers did not describe the same level of conflict between employment and caregiving.

This disparity was further emphasized as caregivers who remained employed reported that the competing demands of work and caregiving created additional barriers to accessing formal support services. Participants described how the dual role of worker and caregiver constrained their ability to engage with available respite programs and support groups, particularly when these services were scheduled during standard working hours. Consequently, even when supportive resources were available, structural factors such as inflexible work schedules and program timing limited their practical accessibility. One participant expressed concern about the increasing care needs of their loved one in the context of full-time employment, noting, “*I do worry that the more support he needs, and with me working all the time… I mean, the job also doesn’t leave a lot of time, right, for that kind of thing, if they’re going to meet on a day that I’m working, well, you know*” (CG1). Similarly, another caregiver observed that available support programming appeared better suited to retirees than working individuals, explaining, “*I did feel like [the support group] was geared to people that are retired already. And so, the timing wasn’t there. And I don’t have a lot of extra time right now in my life*” (CG7). This perception suggests that support services may inadvertently exclude working caregivers when scheduling and delivery models fail to account for employment-related constraints. Collectively, these findings underscore how systemic and organizational barriers, including the alignment of service provision with traditional work hours, may reduce equitable access to respite and support among employed caregivers.

#### 3.1.2. The Financial Burden of Parkinson’s Disease: Alleviating Stress and Enhancing Support Through Economic Stability

Across participants, the financial impact of PD was described as substantial and ongoing, with costs often increasing as the condition progressed. Expenses related to medications, therapies, home modifications, transportation, and potential transitions to safer housing or long-term care created significant economic pressure for many families. For some caregivers, these costs represented a major disruption in household finances and required difficult decisions about resource allocation. Participants frequently described the recurring costs associated with supportive services, with one caregiver noting, “*She was involved with [exercise group]… but they got very very expensive, which is probably the one recurring theme we find with Parkinson’s support groups*” (CG5). As care needs increased, financial strain often extended to larger structural changes, such as modifying living arrangements to ensure safety. One participant described how financial limitations complicated necessary housing transitions, stating, “*we just don’t have the money to just pack up and move*” (CG4). Concerns about future care expenses were even more pronounced, particularly regarding intensive or long-term support, as another caregiver emphasized “*if you had to hire 24/7 care… you’re looking at over $150,000… Way out of most people’s reach*” (CG6).

Economic resources appeared to shape the caregiving experience. Caregivers with greater financial stability reported more flexibility in accessing services, supports, and respite, which helped mitigate stress and preserve well-being. For example, one caregiver explained, “*… I started hiring care workers to give me more time while I went out for lunch with friends, it was another hundred bucks on top of my lunch bill to get out… which gets expensive… but I spent the money anyway because I needed to*” (CG4). In contrast, caregivers facing financial constraints often described having to forgo or delay beneficial programs, activities, or support services due to affordability concerns. One participant emphasized this limitation, stating, “*There’s a bunch of stuff I can enroll him in, but can we afford 90 percent of it? No… With me as the only real income and the cost of living and housing being where it’s at right now? No*” (CG1). Financial hardship also compounded broader life stressors, as this same caregiver further explained: “*I’ve got, you know, my own stresses with work and trying to get my life in order and, you know, I’m in all kinds of debt. We’re just I’m just trying to keep him afloat, and it’s been just a total scramble, trying to keep both of our lives moving*” (CG1). These accounts illustrate how limited economic resources often intensified emotional distress and caregiver burden by restricting access to external support systems.

A recurring tension emerged between maintaining personal well-being and managing financial demands. While paid respite and support services were recognized as important for sustaining caregivers’ mental health, their associated costs often introduced additional stress. Some participants described making difficult trade-offs, often prioritizing the care recipients’ needs over their own. As one caregiver stated, “*if it’s going to cost me money, I certainly can’t afford it… I’m worried about getting him back in boxing. I’m not worried about my own crap right now*” (CG1). This pattern highlights how financial pressures could lead caregivers to deprioritize self-care in favor of preserving resources for the person living with PD.

Overall, economic stability emerged as a key determinant of caregivers’ capacity to access support, maintain personal well-being, and sustain caregiving over time. Financial hardship often compounded the broader emotional and practical burdens associated with PD caregiving, whereas greater economic resources provided flexibility that could alleviate stress and enhance caregiving sustainability.

### 3.2. Theme 2: Psychological and Emotional Dynamics of Caregiving

This theme captures the emotional and relational dimensions of male caregiving, shaped by gendered expectations, approaches and attitudes to living with the illness adopted by both caregivers and IwPD, and ongoing experiences of loss. Participants described caregiving as not only a practical responsibility but also a psychologically complex role that involved negotiating self-perceptions, relationship dynamics, and shifting identities over time. Three subthemes emerged within this theme.

#### 3.2.1. Male Caregivers’ Self-Doubt and Beliefs in Women’s Natural Aptitude for Providing Care

Many caregivers expressed beliefs that women were inherently better suited to caregiving roles, often framing caregiving as more naturally aligned with traditionally feminine traits such as empathy, patience, and attentiveness. These perceptions reflected deeply internalized gender norms surrounding care work and emotional labour, shaping how male caregivers understood both the caregiving role itself and their own performance within it. Several participants suggested that women possessed an innate advantage in providing care, particularly in relation to emotional responsiveness and attentiveness. As one caregiver reflected, “*I’m sure if I were the patient and she was the caregiver, she would be better at it… I think that women, by their very nature, are probably more caring and probably do a better job*” (CG2). Similarly, another participant emphasized the perceived ease with which women manage intimate and day-to-day caregiving tasks, explaining, “*Women seem to be more easily empathetic about all of that sort of thing than men are… I think, it comes more easily to the woman than it does for the man*” (CG4).

These perceptions reflected internalized gender norms about care work and emotional labour. Several participants suggested that caregiving tasks involving personal or intimate care felt less natural or comfortable for them, reinforcing the belief that women possessed a greater innate capacity for such responsibilities. This sense of gendered expectation shaped how some men evaluated their own performance as caregivers and contributed to feelings of uncertainty or lowered confidence in their caregiving role.

For some male caregivers, these gendered assumptions contributed to feelings of self-doubt, perceived inadequacy, and uncertainty regarding their caregiving abilities. Tasks involving personal or intimate care were sometimes described as feeling less natural, reinforcing beliefs that men were less inherently equipped for caregiving responsibilities. One participant explicitly acknowledged this perceived disparity, stating, “*If the roles were reversed, she would be far stronger at it all the way down the line than I may be*” (CG5). Another caregiver similarly noted, “*I do have a stereotype that probably more females are oriented to caregiving… I think that’s partly… culturally… to be a male in that role is a bit different*” (CG6). These reflections suggest that caregiving was often viewed through a gendered lens, where male caregivers perceived themselves as operating outside conventional social expectations.

This internalized sense of gendered expectation also appeared to shape emotional experiences within caregiving. Some participants described heightened anxiety, stress, and persistent self-questioning about whether they were adequately fulfilling their caregiving responsibilities. One caregiver described this ongoing psychological burden, stating, “*You get as a caregiver, you get depressed, you get stressed, you get worried that you’re not doing the right thing… Find yourself waking up at 3:00 in the morning… thinking did I handle that conversation right?*” (CG4). Collectively, these findings illustrate how traditional gender norms not only influenced perceptions of caregiving competence but also contributed to emotional strain, lowered confidence, and increased psychological burden among male caregivers.

#### 3.2.2. The Power of Perspective: Patient and Caregiver Attitudes Influencing the Care Journey

Participants described how the outlook and coping approaches of both the caregiver and the IwPD shaped the overall caregiving experience. A shared focus on acceptance, problem-solving, and maintaining a positive outlook was often described as helping couples navigate the challenges associated with the diagnosis and disease progression. Caregivers noted that when the IwPD adopted a pragmatic or resilient attitude, it reduced emotional strain and fostered a sense of shared purpose. One caregiver explained, “*I think a lot of it has to do with the attitude of the person with the disease as to how it is going to affect or tie into how the caregiver or care partner feels…, it’s sort of a shock to get the diagnosis that she did. But at the same time, she had the attitude of, OK, what do I have to do… never once was it, well, why me? … Why should I feel sorry for myself as a partner if she doesn’t feel sorry for herself because she has this disease?*” (CG3).

Caregivers also emphasized the importance of their own mindset, highlighting efforts to avoid dwelling on negative outcomes and instead focus on day-to-day coping. Maintaining a hopeful or balanced perspective was described as a strategy for sustaining emotional well-being and preserving relationship stability in the face of ongoing uncertainty. As one participant noted, “*Don’t deal with it passively. Don’t turn yourself into another victim. And even the person with Parkinson’s is far better off if they can get out of that victim mindset. And the caregiver is better if they stay out of that victim mindset. And that’s not easy*” (CG6). Similarly, another caregiver described the importance of limiting focus on uncertain future scenarios, explaining, “*You can spend a whole lot of time on the what-ifs. And if the what-ifs never happen, you’ve wasted all that energy and all that stress and everything else…I think we’ve sort of come to the conclusion together. It’s let’s take it a day at a time, and that’s the only thing you can control right now*” (CG3). Together, these accounts illustrate how caregivers actively worked to regulate their outlook, using present-focused coping and rejection of a “victim mindset” to manage stress and maintain resilience.

Beyond individual attitudes, participants discussed how decisions about disclosing the diagnosis influenced social and relational experiences. When IwPD were open about their condition, caregivers reported greater access to understanding social networks and informal support. As one person shared, “*I’m very fortunate. I have a couple of friends that I can and have sort of reached out to because they at least understand… They understand her PD; they understand that there are good days and bad days. They’re a great group. And I think some of their support, whether they know it or not, is very important*” (CG5).

In contrast, when the diagnosis was kept private, caregivers described increased isolation and reduced opportunities for assistance from friends, family, or community members. One caregiver explained how a preference for privacy limited external support and social engagement: “*The difficulty I’ve really been struggling with the last year is [Wife’s name] is the introvert of us, and she’s been wanting to be very private about her struggles… I offered a couple of retired friends, women, who could come and sit with her, which almost horrified her… And I try to reassure that people understand, they genuinely care for her… but [Wife’s name] tells me so many times, don’t tell anybody about this… [We’re] losing a lot of social outlets… it’s been 4 or 5 years since she’s gone with me [to church] and visiting*” (CG7). This dynamic created additional emotional strain and raised concerns about where caregivers could turn for support during difficult periods. As another participant reflected, “*where does the caregiver turn to when you start running out of runway and you’re frustrated, what do you do to deal with the situation, or it has been a rough week. Who do you call to talk to about that?*” (CG2).

#### 3.2.3. Living with Loss: Enduring Grief from the Gradual Erosion of Normalcy

Participants described caregiving as accompanied by an ongoing experience of loss, characterized by the gradual erosion of shared roles, activities, and relationship dynamics. Rather than a single moment of grief, caregivers portrayed loss as a continuous process unfolding alongside disease progression. As functional abilities declined, couples experienced changes in routines, independence, and previously shared interests, which altered the nature of their relationship. As one caregiver expressed, “*I’ve lost my bright, physically able partner who could go with just the two of us and we could manage our sailboat. We could go on ski trips, we could go on a car trip, share driving, etc. You have the inescapable sense that there’s something that’s been taken away. And that aspect of it never goes away*” (CG6).

Caregivers reported that this process involved repeated adjustments to new limitations, often accompanied by emotional responses resembling a stage-based grieving process. Each decline in function or change in daily life represented another loss, requiring psychological adaptation. These losses were not only physical but also symbolic, affecting identity, intimacy, and the sense of partnership that had existed before the illness. One participant described this cumulative experience, noting, “*As things get worse, as things disappear… there’s a real sense of loss when the sort of restrictions that go with age are accelerated by the disease of your partner. And, you know, you both feel what is being lost, and there’s nothing you can do about it. And that’s very frustrating*” (CG6).

Everyday transitions, such as changes in mobility, sleeping arrangements, or driving status, were described as emotionally significant markers of disease progression. These moments served as tangible reminders of irreversible change and reinforced the ongoing nature of grief within the caregiving experience. For example, one caregiver reflected on how seemingly practical decisions carried emotional weight, explaining, “*She hasn’t been behind the wheel in the car in at least three years now… when our insurance was going to renew, I asked, do you want to take your name off the insurance? But that turned out to be very hurtful because then it became official that she was never going to drive again… And we talked a lot about our sleeping situation… I said shall we go to the twin beds… but if we get rid of our king-size mattress, she knows we won’t be going back to that bed… and many things like that. And so, there’s, in a way, those are all losses and forms of grieving*” (CG7). Overall, caregiving was characterized by a persistent undercurrent of mourning for the life, roles, and relationship dynamics that had gradually been altered or lost.

### 3.3. Theme 3: Adaptation and Coping in Caregiving

This theme captures how caregivers recognized the necessity of adapting to the evolving demands of PD, often striving to preserve pre-diagnosis routines while modifying them to minimize disruption. Caregiving was described as a dynamic process requiring ongoing adjustments in daily activities, household roles, and emotional support strategies. Coping approaches varied across participants. For some, actively seeking information about PD was experienced as empowering and provided a sense of direction, whereas for others, information seeking elicited anxiety and apprehension about potential future decline. Two subthemes emerged within this theme.

#### 3.3.1. Embracing New Realities: Navigating New Roles and Increased Responsibilities

Participants described the gradual renegotiation of shared roles and responsibilities as a central aspect of adapting to caregiving. Many sought to preserve familiar routines and activities, modifying them as needed to accommodate changes in physical or cognitive functioning. As one caregiver explained, early strategies involved supporting continued participation through environmental cues: “*I put labels on the stove, on the washing machines… to sort of you know, first to do this, to do this sort of thing. That helped for a while. And then you get to a point where no, the person shouldn’t be doing it anymore. The caregiver has to do it*” (CG6). Others described adapting shared activities to maintain connection while accommodating differing capacities: “*We still go for walks basically three or four times a week. [My wife] usually does a shorter walk, and I’ll continue on for another hour after that*” (CG3). Together, these accounts illustrate how couples attempted to maintain a sense of continuity and shared identity, even as daily life shifted.

At the same time, caregivers frequently assumed new household and practical responsibilities that had previously been shared or primarily managed by their partner. This transition often involved learning unfamiliar tasks and taking on a greater share of domestic labour, contributing to what participants described as part of adjusting to a “new normal.” One caregiver conveyed the abruptness and breadth of this shift: “*Laundry was one. Shopping is another. Housekeeping is another. How the hell does the vacuum cleaner work? Doing the dishes either manually or through a dishwasher. All of those things. Just the running of the household… all those things immediately drop in your lap*” (CG4). Despite these challenges, some participants identified opportunities for collaborative adaptation, reframing tasks as shared activities: “*We’ve actually made a new routine these last months… We make supper together now on Saturday afternoons, which involves her giving directions and me making supper. So, it’s been mutually beneficial because I’ve learned a lot and it’s a nice thing to be able to do together*” (CG7). These narratives underscore both the disruption and potential for reconfigured partnership with evolving domestic roles.

Beyond practical adjustments, caregivers also described taking on increased emotional and advocacy responsibilities. This included supporting the IwPD through difficult moments, navigating healthcare interactions, and speaking on their behalf when necessary. While these roles were viewed as important aspects of caregiving, they were also associated with emotional strain. One caregiver articulated the profound personal impact of these responsibilities: “*My life is now on hold, and like my whole life plan is now basically out the window, and I’ve basically dedicated my future to him… I’ve kind of accepted that this is my life now, this is where we’re at, and this is where I’m at until he goes to a home, and that’s just the way it is*” (CG1). Caregivers also consistently reported feelings of stress, worry, and uncertainty, often intensified by the progressive and incurable nature of the illness, as reflected in the observation that “*These feelings can be compounded by the knowledge that there is no cure to PD, and the illness itself psychologically, it’s something tough to deal with because there’s no solution to it*” (CG2). In addition, caregivers emphasized the relational and communicative demands of their role, highlighting the need to develop new interpersonal skills: “*You have to learn how to listen. You have to learn how to ask sometimes slightly impertinent questions. You have to learn how to engage with what’s going on*” (CG6). Overall, adaptation involved not only changes in daily routines but also a significant shift in emotional and relational responsibilities.

#### 3.3.2. Pursuing Knowledge: A Pathway to Control or a Gateway to Fear

Caregivers frequently described a lack of formal guidance or structured training in their caregiving role. Many characterized the experience as one of “learning on the job,” with limited direction from formal systems of care. This absence of clear information and support contributed to feelings of uncertainty, isolation, and trial-and-error approaches. As one participant explained, “I’ve kind of felt like I’ve been on my own this whole time, just kind of fumbling in the dark, trying to keep us afloat… I’m just flailing in the water (CG1). Another similarly emphasized the extent to which they depended on informal sources of knowledge: “if it hadn’t been for [wife’s] research, we’d be waddling around like a couple of rubber ducks bobbing in the Detroit River” (CG5).

At the same time, participants expressed mixed experiences with information seeking. For some, acquiring knowledge about PD was perceived as empowering, providing a sense of preparedness and control over an otherwise unpredictable situation. Access to reliable information was seen as a way to reduce helplessness and better anticipate future needs. As one caregiver noted, “*Listening to the people who are actually doing the work and listening to the people who are suffering from the disease… helps you to know what’s coming and what’s in the future for you*” (CG4). Similarly, another participant highlighted the value of synthesized evidence: “*But if you could come across a paper that was doing a sort of meta-analysis of what the literature was saying at any given point, that’s very useful… being informed gives you, maybe a false one, but some sense of control. You don’t feel as totally helpless as you might otherwise*” (CG6).

In contrast, other caregivers described ambivalence or avoidance regarding information seeking. Exposure to information about disease progression and potential complications was sometimes experienced as emotionally overwhelming, leading some participants to limit engagement with available resources. For example, one caregiver described avoiding exposure to more advanced stages of the illness, noting that “*she probably would never, ever want to meet [Friend’s husband with advanced Parkinson’s] because she knows what’s coming and she doesn’t want to think about that. And so not wanting to dwell on how awful it may get at the end*” (CG7). Another participant similarly reflected on withdrawing from a support group due to the distress associated with observing disease progression in others: “*We tried to go to a local Parkinson’s support group a few times when she was diagnosed… she found it depressing just watching people how they have progressed farther, they might be 5–10 years ahead of her in their illness and we haven’t kept going to that*” (CG2). This created a tension between the desire for knowledge and the fear of confronting distressing possibilities. As one caregiver reflected, “*It’s a weird balance, I should be researching it more, but at the same time, I don’t want to because it’s scary stuff… I don’t want to hear that kind of stuff, I could be dealing with later…*” (CG01).

Overall, information seeking functioned as both a coping strategy and a potential source of anxiety, highlighting the complex emotional landscape of caregiving adaptation. Together, these themes illustrate caregiving as a dynamic process shaped by structural constraints, evolving relational roles, and ongoing emotional and practical adaptation.

## 4. Discussion

This study provides in-depth qualitative insights into the experiences of male informal caregivers, an understudied group in PD research. The findings portray caregiving as a multifaceted process shaped not only by disease-related demands but also by structural and economic pressures, gendered role expectations, and the need for continual adaptation as roles and relationships evolve. Together, the results suggest that male caregiving in PD is shaped by the intersection of financial constraints, relational dynamics, and shifting identities over time. By focusing specifically on male caregivers, this study extends existing PD caregiving literature, which has historically centered on female caregivers or mixed-gender samples with a predominance of female caregivers [17].

### 4.1. Economic and Employment Challenges in Caregiving

Consistent with Theme 1, participants described caregiving as embedded within broader economic and employment contexts. Within this theme, a subset of participants—primarily those working full time—reported difficulty balancing employment with caregiving responsibilities, often viewing work as a financial necessity rather than a choice. Caregivers described challenges reducing work hours or accessing supports that conflicted with employment schedules, resulting in fatigue and limited opportunities for respite. These findings align with previous research by Gallop et al. [31], which reported that caregivers of people with PD often needed to reduce work hours or leave employment entirely in order to provide care. These experiences reflect broader evidence that Parkinson’s caregivers often provide levels of informal care comparable to a second full-time job as disease-related needs increase [32].

Participants also described the substantial financial burden associated with PD, particularly as the disease progresses. Economic resources shaped caregivers’ ability to access programs, respite, and supportive services, with financial stability acting as a buffer against stress and economic precarity intensifying caregiving demands. Prior research has documented higher out-of-pocket and indirect costs among Parkinson’s caregivers, as well as cumulative income loss over time [33]. Expanding on these findings, Gallop et al. [31] identified reduced net income due to fewer working hours alongside increased expenses related to medications and medical equipment, such as walkers and wheelchairs, as key contributors to caregiver financial strain. National data further indicate that financial hardship is especially common among caregivers providing higher hours of unpaid care, and that financial security is the most frequently identified support need [32]. These findings are consistent with broader evidence indicating that caregivers with greater financial resources tend to experience lower levels of burden, whereas financial strain and lower socioeconomic status are associated with poorer caregiver outcomes [34]. This is particularly important given emerging evidence that lower-earning caregivers experience less favourable health trajectories even after active caregiving has ended [35]. Together, these findings highlight the structural tension between employment and caregiving and underscore the importance of economic supports that enable access to respite and formal services.

### 4.2. Psychological and Emotional Dynamics of Caregiving

Aligned with Theme 2, the findings highlight the psychosocial and relational dimensions of caregiving. Many participants viewed caregiving as a feminized role, contributing to self-doubt and beliefs that women were inherently more capable caregivers. These findings align with gender role theory and research on hegemonic masculinity, which position caregiving as incongruent with dominant masculine ideals and may heighten self-doubt among men [36].

At the same time, prior research suggests that overall gender differences in caregiver stressors and outcomes are often small [22], indicating comparable levels of burden across male and female caregivers. Such differences may be better explained by prior role socialization than by inherent gender-based caregiving abilities. Women who have historically managed household and caregiving tasks may enter caregiving roles with more established routines and coping strategies, whereas men may face a steeper learning curve and have fewer preexisting skills, as suggested by comparative analysis of older male and female caregivers [37].

This pattern was reflected in participants’ accounts of rapidly acquiring new competencies and assuming responsibilities previously managed by their partners. Together, these findings highlight the importance of providing male caregivers with targeted, practical, and psychosocial supports that address both skill development and gendered barriers to adaptation.

Participants also described how the attitudes of both caregivers and IwPD shaped the caregiving experience. Acceptance-oriented, problem-focused approaches were associated with greater emotional stability and a sense of shared resilience, whereas avoidant coping was linked to increased strain. These findings align with prior research showing that acceptance-based coping and positive illness appraisals are associated with lower caregiver burden, partly through increased perceived social support [5]. The relational context of caregiving was further shaped by disclosure practices. When the diagnosis was kept private, caregivers experienced constrained access to social support, increased isolation, and reduced opportunities to seek both emotional and practical assistance. These restrictions limited caregivers’ ability to explain changes in functioning, justify the need for help, or draw on informal networks, thereby increasing the emotional and practical burden of caregiving. This is particularly important given evidence that social isolation and reduced social support are key contributors to caregiver burden in PD [9,17].

Caregiving was also characterized by ongoing experiences of loss, reflecting the gradual erosion of shared roles, activities, and a sense of normalcy. Participants described progressive changes in relationship dynamics and daily routines, suggesting that caregiving involved repeated emotional adjustments rather than a single period of adaptation. These findings align with prior work demonstrating that shifts in roles from spouse to caregiver can fundamentally alter relationship dynamics and contribute to feelings of sadness, loss, and changes in caregiver identity, as well as the loss of future plans and shared expectations [9]. These cumulative losses are consistent with the literature on anticipatory grief [38]. Recognizing grief as a sustained and normative aspect of caregiving may help normalize caregivers’ experiences and inform interventions that address emotional well-being across the disease trajectory.

### 4.3. Adaptation and Coping in Caregiving

Reflecting Theme 3, caregiving emerged as an adaptive process requiring the renegotiation of roles, routines, and responsibilities. Caregivers described maintaining familiar activities where possible while assuming new practical and emotional responsibilities, including household management and advocacy within healthcare settings. These experiences are consistent with prior research conceptualizing caregiving as a dynamic process involving ongoing role transitions and adjustments as illness progresses [17,39]. Although such adaptations may help preserve continuity and partnership, they were also associated with emotional strain, particularly given the progressive nature of PD and the absence of curative treatments [17].

The accounts also revealed the complex role of information seeking in coping. Many caregivers reported limited formal guidance and described caregiving as a process of “learning on the job.” Previous work by Hulshoff and colleagues [40] similarly identified the need for information as a key theme in caregiver experiences, with information needs subdivided into five subthemes: prognostic information for future planning, Parkinson’s disease treatment, care partner education resources, finances, and home adaptation. In the present study, some participants also described how acquiring information fostered a sense of control and preparedness, whereas for others, information about disease progression elicited fear, creating tension between the desire for knowledge and the emotional cost of confronting uncertain futures. This dual role of information as both a resource and a stressor has been noted in prior caregiving research, which suggests that access to information can enhance caregiver self-efficacy while also increasing distress when it highlights future decline or uncertainty [41,42]. Together, these findings underscore the importance of tailored, stage-appropriate education and support.

### 4.4. Limitations

These findings should be interpreted in light of several limitations. First, the relatively small sample size and recruitment from a regional PD not-for-profit organization may limit the transferability of the findings to broader caregiver populations or different geographic and service contexts. Second, the three participants in phase one were not specifically asked to reflect on caregiving as a gendered experience, which may have resulted in less detailed or less comparable insights on this topic relative to other participants. In addition, one participant in phase 2 declined to participate in a follow-up interview, which limited the opportunity for member checking and participant validation of emerging interpretations in that case. Finally, no standardized measures of caregiver burden were collected. This limited the ability to systematically compare experiences across levels of caregiving intensity. Together, these factors should be considered when interpreting the scope and applicability of the findings.

### 4.5. Implications and Future Directions

Findings from this study underscore the need for caregiver education and support resources that are explicitly tailored to disease stage and the evolving demands across the trajectory of PD. Stage-appropriate resources should provide practical guidance on managing motor and non-motor symptoms, navigating care transitions, accessing community and home-based supports, and planning for financial and legal considerations, with content calibrated to early, middle, and advanced stages. Embedding these resources within routine clinical pathways, such as movement disorder clinics, community PD organizations, and primary care, may help mitigate cumulative caregiver strain and reduce reliance on reactive, crisis-driven supports.

These findings further highlight the need to prioritize caregiver well-being as an integral component of PD care. Resources must extend beyond caregiving task-oriented support to address the psychological and emotional demands associated with sustained caregiving. Without adequate support, caregiver distress and burnout may compromise the sustainability of care and contribute to increased system-level healthcare burden [43].

Within this context, cognitive behavioural therapy represents a promising, evidence-based approach that has been found to effectively improve psychological outcomes such as depression, anxiety, coping, and self-efficacy [44,45,46]. In this regard, our research team is currently adapting an internet-based cognitive behavioural therapy program to better meet the specific needs of caregivers supporting IwPD. This work involves tailoring an existing evidence-based program [47] to reflect the lived experiences and challenges of PD caregiving, including managing stress, addressing unhelpful thought patterns, maintaining daily functioning, and supporting overall quality of life.

Next steps include evaluating the adapted program in a pilot study to assess its feasibility, acceptability, and preliminary efficacy. By equipping caregivers with practical coping strategies and psychological tools, this future research aims to reduce caregiver stress and burnout, strengthen emotional resilience, and ultimately support the provision of high-quality, sustainable care, with potential downstream benefits for IwPD.

More broadly, future research in this domain should incorporate standardized measures of caregiver burden and well-being to enable consistent evaluation across studies, facilitate comparisons across different levels of caregiving intensity, and support the identification of caregivers at heightened risk of distress or burnout. Such measures would allow for more precise assessment of intervention effects and inform the tailoring of supports to better align with caregivers’ evolving needs over time. In addition, future work should include more diverse caregiving populations to enhance generalizability and examine implementation strategies such as integration within clinical pathways and partnerships with community organizations to support the scalability, accessibility, and sustainability of evidence-based caregiver interventions.

## 5. Conclusions

The findings of this study have important implications for clinical care and service delivery for male caregivers supporting IwPD. Results highlight the need for gender-responsive, integrated care models that address financial barriers to respite and formal supports, promote flexible workplace accommodations, and provide practical training and psychosocial interventions to address self-doubt, coping, and grief. For movement disorder clinics and multidisciplinary teams, these findings underscore the importance of routinely assessing caregiver needs, facilitating access to social and financial supports, and incorporating dyadic approaches that support shared adjustment and communication. Together, these implications emphasize the need for scalable, system-level interventions that address both structural and psychosocial determinants of caregiver well-being and optimize care for IwPD, for whom caregivers play a central and often indispensable role in ongoing management and support.

## Figures and Tables

**Table 1:** Participant demographic characteristics.

Participant ID	Age	Relationship to IwPD	Time Since Diagnosis	Employment Status	Living Situation
CG1	26	Son	6 years	Full Time	At home with parents
CG2	NR ^1^	Married 49 years	10 years	Retired	At home with spouse
CG3	71	Married 47 years	9 years	Part Time	At home with spouse
CG4	90	Married 65 years	10 years	Retired	Spouse in long-term care
CG5	NR ^1^	Married NR years	8 years	Retired	At home with spouse
CG6	88	Married 56 years	19 years	Retired	Widow (living alone)
CG7	NR ^1^	Married 39 years	8 years	Full Time	At home with spouse

^1^ NR = Not Reported.

## Data Availability

The qualitative data generated and analyzed during the study are not publicly available because they contain potentially identifiable participant information and participants did not consent to public data sharing. De-identified excerpts supporting the findings are included within the article. Additional information may be available from the corresponding author upon reasonable request and subject to institutional ethics approval.

## Abbreviations

The following abbreviations are used in this manuscript:

PD	Parkinson’s disease
IwPD	Individuals with Parkinson’s disease
NR	Not reported
CG	Caregiver
